# Supplementary material for: Long Non-Coding RNA MIR31HG Promotes the Transforming Growth Factor β-Induced Epithelial-Mesenchymal Transition in Pancreatic Ductal Adenocarcinoma Cells
Source: Int J Mol Sci. 2022 Jun 12;23(12):6559. doi: 10.3390/ijms23126559 (PMC9223781; doi:10.3390/ijms23126559)
Supplement: Supplementary file 1 [file ijms-23-06559-s001.zip › ijms-1735368-supplementary.pdf]

<u>**Supplementary Information**</u>

# Long Non-Coding RNA *MIR31HG* Promotes the Transforming Growth Factor β-Induced Epithelial-Mesenchymal Transition in Pancreatic Ductal Adenocarcinoma Cells

**Ching-Chung Ko [1,2,3], Yao-Yu Hsieh [4,5] and Pei-Ming Yang [6,7,8,9,10,*]**

1 Department of Medical Imaging, Chi Mei Medical Center, Tainan 71004, Taiwan; kocc0729@gmail.com
2 Department of Health and Nutrition, Chia Nan University of Pharmacy and Science, Tainan 71710, Taiwan
3 Institute of Biomedical Sciences, National Sun Yat-Sen University, Kaohsiung 80424, Taiwan
4 Division of Hematology and Oncology, Taipei Medical University Shuang Ho Hospital, New Taipei City 23561, Taiwan; alecto39@gmail.com
5 Division of Hematology and Oncology, Department of Internal Medicine, School of Medicine, College of Medicine, Taipei Medical University, Taipei 11031, Taiwan
6 Graduate Institute of Cancer Biology and Drug Discovery, College of Medical Science and Technology, Taipei Medical University, Taipei 11031, Taiwan; yangpm@tmu.edu.tw
7 PhD Program for Cancer Molecular Biology and Drug Discovery, College of Medical Science and Technology, Taipei Medical University and Academia Sinica, Taipei 11031, Taiwan
8 TMU Research Center of Cancer Translational Medicine, Taipei 11031, Taiwan
9 Cancer Center, Wan Fang Hospital, Taipei Medical University, Taipei 11696, Taiwan
10 Taipei Medical University (TMU) and Affiliated Hospitals Pancreatic Cancer Groups, Taipei Cancer Center, Taipei Medical University, Taipei 11031, Taiwan
* Correspondence: yangpm@tmu.edu.tw; Tel.: +886-2-2697-2035 (ext.143)

**Supplementary Information** includes:

Figure S1: The uncropped images of the Western blots.

# Full unedited blot for Figure 3C

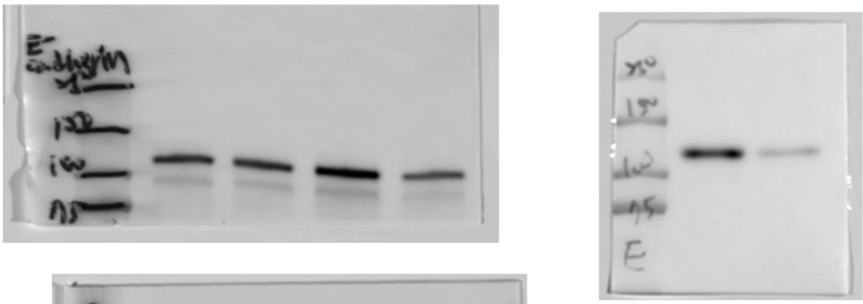

E-cadherin (CDH1)

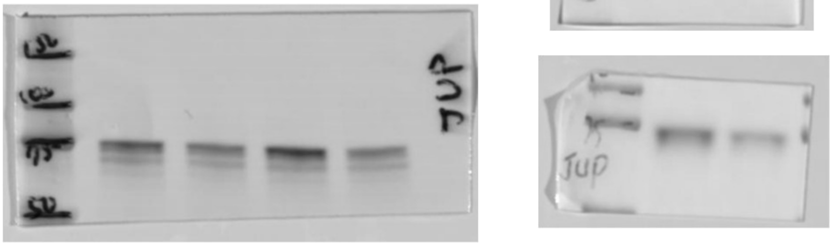

Plakoglobin (JUP)

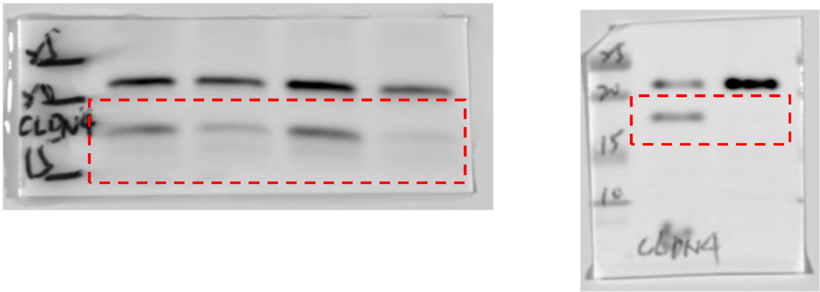

Claudin 4 (CLDN4)

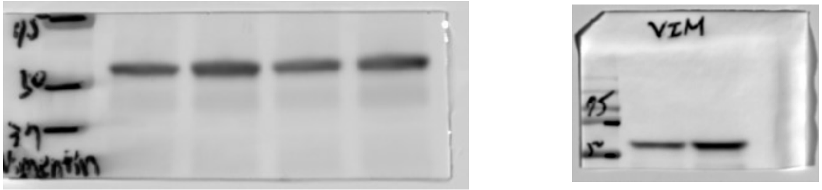

Vimentin (VIM)

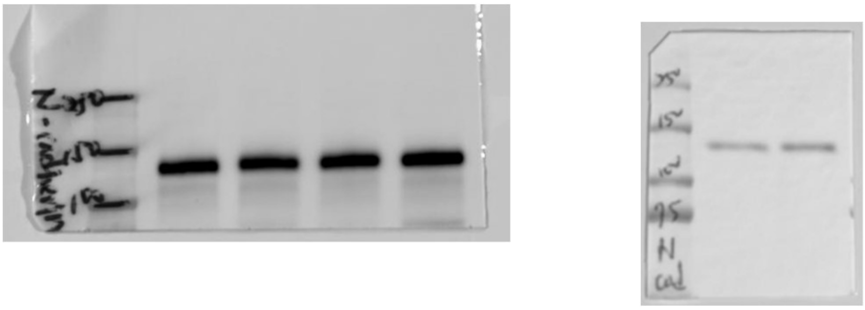

N-cadherin (CDH2)

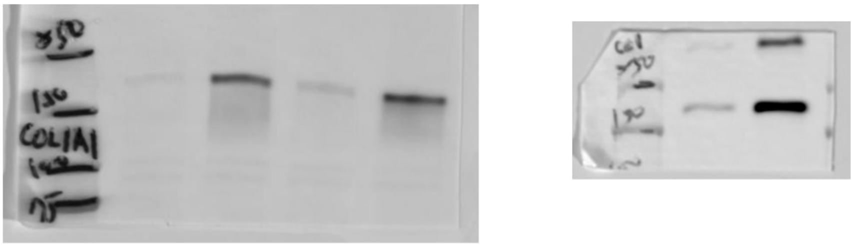

Collagen 1A1 (COL1A1)

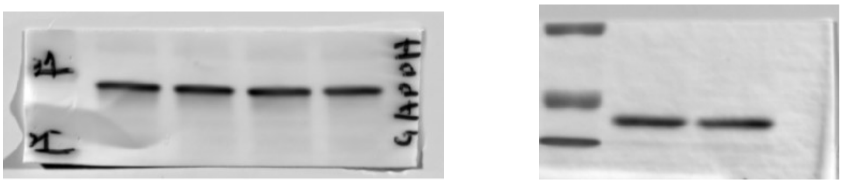

GAPDH

# Full unedited blot for Figure 4C

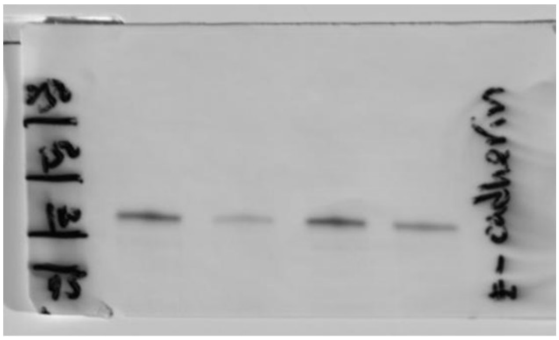


E-cadherin (CDH1)

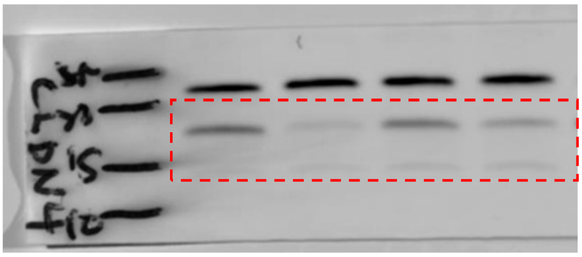


Claudin 4 (CLDN4)

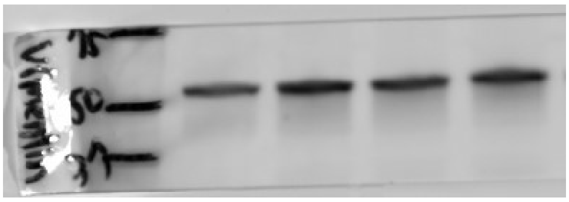


Vimentin (VIM)

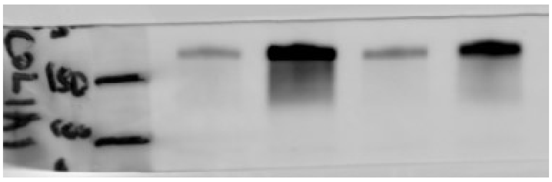


Collagen 1A1 (COL1A1)

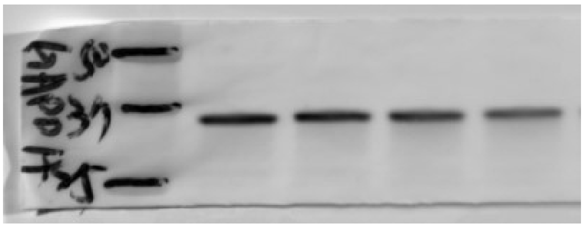


GAPDH

# Full unedited blot for Figure 4F

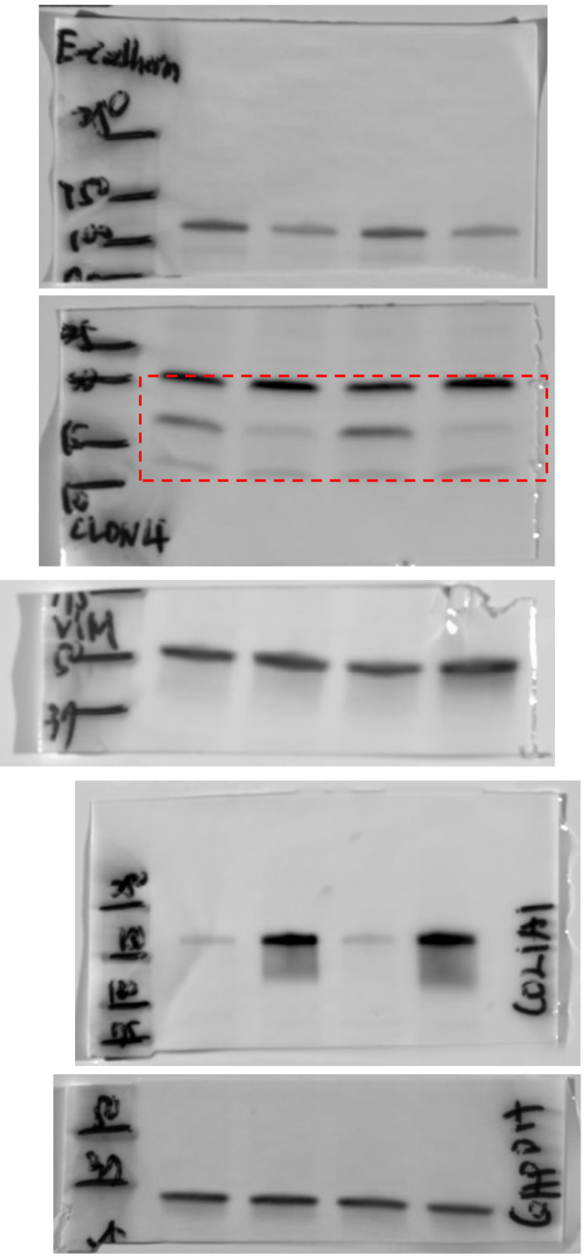


E-cadherin (CDH1)

Claudin 4 (CLDN4)

Vimentin (VIM)

Collagen 1A1 (COL1A1)

GAPDH
